# ERVWE1 Impairs Mitochondrial Homeostasis and Promotes Neuronal Apoptosis via the miR-27b-3p/BNIP3 Axis in Schizophrenia

**DOI:** 10.3390/v18020245

**Published:** 2026-02-14

**Authors:** Yaru Su, Kexin Zhao, Mengqi Zhang, Jiahang Zhang, Zhao Lv, Fangyi Hou, Xu Zhang, Zhao Zhang, Fan Zhu

**Affiliations:** 1 State Key Laboratory of Virology and Biosafety, Department of Medical Microbiology, School of Basic Medical Sciences, Wuhan University, Wuhan 430071, China; 2 Hubei Province Key Laboratory of Allergy and Immunology, Department of Medical Microbiology, School of Basic Medical Sciences, Wuhan University, Wuhan 430071, China

**Keywords:** ERVWE1, BNIP3, miR-27b-3p, mtDNA, apoptosis

## Abstract

Schizophrenia is a severe neurodevelopmental disorder with a complex and largely unresolved pathogenesis. Accumulating evidence indicates that mitochondrial dysfunction is a consistent pathological hallmark of schizophrenia, suggesting that impaired mitochondrial homeostasis may represent a convergent mechanism underlying disease vulnerability. BCL2/adenovirus E1B 19 kDa interacting protein 3 (BNIP3) is a critical regulator of mitochondrial integrity and apoptosis. However, its role in schizophrenia has not yet been elucidated. Human endogenous retroviruses W family envelope (ERVWE1) has been implicated as a potential risk factor in schizophrenia, but the molecular mechanisms by which it contributes to neuronal pathology remain poorly understood. In this study, we investigated whether ERVWE1 induces mitochondrial dysfunction and neuronal apoptosis through the regulation of BNIP3. Bioinformatic analysis of the public dataset GSE53987 revealed significantly elevated *BNIP3* expression in the brain tissues of patients with schizophrenia, accompanied by enrichment of mitochondria-related pathways. Consistently, BNIP3 expression was also increased in the peripheral blood of schizophrenia patients and positively correlated with ERVWE1 levels. Mechanistically, ERVWE1 upregulated BNIP3 expression by suppressing miR-27b-3p, a microRNA that directly targets BNIP3. The resulting increase in BNIP3 led to marked mitochondrial structural and functional impairment, characterized by reduced mitochondrial aspect ratio, enhanced mitochondria permeability transition pore (mPTP) opening, and decreased mitochondrial DNA (mtDNA) copy number. These mitochondrial defects subsequently triggered cytochrome c release into the cytosol, activating the intrinsic mitochondrial apoptotic pathway. Collectively, this study provides the first evidence that the ERVWE1/miR-27b-3p/BNIP3 axis contributes to mitochondrial dysfunction and neuronal apoptosis in schizophrenia. Our findings identify a previously unrecognized molecular pathway linking endogenous retroviral activity to mitochondrial pathology, offering novel insights into the mechanisms and potential therapeutic targets for schizophrenia.

## 1. Introduction

Schizophrenia is a severe and chronic neuropsychiatric disorder affecting approximately 1% of the global population [1]. Its pathogenesis is highly heterogeneous and involves complex interactions among genetic vulnerability, environmental exposures, and disrupted neurodevelopmental processes [2]. In recent years, advanced experimental models, including brain organoids, have been increasingly applied to recapitulate key aspects of schizophrenia onset and progression [3]. While the classical dopamine hypothesis remains influential [4], growing evidence indicates that additional mechanisms—particularly viral infections [5,6,7] and mitochondrial dysfunction [8]—play critical roles in disease pathophysiology.

Mitochondrial dysfunction has emerged as a central pathological feature not only in neurodegenerative diseases but also in schizophrenia [9,10]. Clinical and postmortem studies have consistently demonstrated both structural [11,12] and functional impairments [13,14] of mitochondria in patients with schizophrenia. These abnormalities disrupt essential cellular processes, including bioenergetic metabolism [15], calcium buffering capacity [16,17], and reactive oxygen species (ROS) homeostasis [18], ultimately compromising neuronal excitability, synaptic transmission, and neural circuit integrity [19]. Severe mitochondrial impairments can further trigger the critical step of initiate cytochrome c release into the cytosol, activating the intrinsic apoptotic pathway and leading to neuronal loss [20].

BCL2/adenovirus E1B 19 kDa interacting protein 3 (BNIP3) is a critical regulator of mitochondrial homeostasis [21] and apoptosis [22,23]. It plays an essential role in mitophagy [24] and the regulation of mitochondrial permeability transition pore (mPTP) opening [25]. Notably, dysregulated *BNIP3* expression has been reported in schizophrenia patients [26]. However, its specific regulatory mechanisms and functional consequences in this disorder remain largely unexplored. BNIP3 expression is known to be modulated by hypoxia-inducible factor 1α (HIF-1α) [27] as well as multiple microRNAs (miRNAs) [28,29,30,31]. MiRNAs are small non-coding RNAs [32] that finely tune gene expression at the post-transcriptional level [33], and widespread miRNA dysregulation has been documented in schizophrenia [34,35]. Among them, miR-27b-3p has been implicated in several neurological disorders [36], yet its potential role in schizophrenia-related mitochondrial dysfunction has not been defined.

Schizophrenia is widely regarded as a disorder arising from the interplay between genetic susceptibility and environmental insults [37,38]. Recent studies have highlighted human endogenous retroviruses (HERVs) as potential molecular intermediaries connecting these two domains [39,40]. HERVs constitute approximately 8% of the human genome and represent remnants of ancient retroviral infections that have been stably integrated into the host DNA [41]. Once considered “junk DNA”, specific HERV elements have since been shown to exert important physiological functions [42], including regulation of immune responses [5]. The *HERV-W env gene*, the envelope of the HERV-W family located on chromosome 7q21.2, encodes the protein ERVWE1 (also called Syncytin-1, ERVW-1), which is essential for placental development and has also been implicated in cancers [43,44], autoimmune diseases [45], and neuropsychiatric disorders [46]. Elevated ERVWE1 expression has been detected in the cerebrospinal fluid, peripheral blood, and brain tissue of schizophrenia patients [9], and its protein product has been linked to neuroinflammation, impaired neurodifferentiation, and synaptic function [47]. Moreover, ERVWE1 appears to act as a molecular bridge between environmental factors, such as drug exposure and neurologic disease susceptibility [48]. Despite these associations, the role of ERVWE1 in neuronal mitochondria and its contribution to mitochondrial structural damage in schizophrenia remain largely unknown.

In the present study, we sought to elucidate the molecular link between ERVWE1 activation and mitochondrial dysfunction in schizophrenia. We first confirmed BNIP3 upregulation and its positive correlation with ERVWE1 expression in clinical samples. Using SH-SY5Y cells, we further demonstrated that ERVWE1 suppresses miR-27b-3p, leading to BNIP3 upregulation, mitochondrial structural and functional impairment, and subsequent activation of the intrinsic apoptosis pathway. Together, our findings uncover a previously unrecognized ERVWE1/miR-27b-3p/BNIP3 signaling axis that connects endogenous retroviral activity to mitochondrial dysfunction and neuronal apoptosis, providing new mechanistic insights and potential therapeutic targets for schizophrenia.

## 2. Materials and Methods

### 2.1. Human Blood Samples

Peripheral blood samples were obtained from Renmin Hospital of Wuhan University, with approval from the Ethics Committee of Wuhan University Medical College. Written informed consent was obtained from all participants prior to enrollment. The schizophrenia group consisted of 44 first-episode patients who met the Diagnostic and Statistical Manual of Mental Disorders, Fifth Edition (DSM-5) criteria and had no history of acute infectious diseases. The control group included 44 healthy volunteers matched for sex, age, body mass index (BMI), education level, and smoking status, and rigorously screened to exclude any neuropsychiatric diseases. Venous blood samples were collected using standardized procedures. Serum was isolated by centrifugation at 1000× *g* for 10 min at room temperature, aliquoted, and stored at −80 °C until further analysis. Clinical sample usage and experimental procedures followed previously established protocols.

### 2.2. Cell Culture and Transfection

The human neuroblastoma cell line SH-SY5Y, provided by the American Type Culture Collection, was used for all in vitro experiments. Cells were cultured in a 1:1 mixture of DMEM (2225320; Gibco, Baltimore, MD, USA) and F12 (2209586; Gibco, Baltimore, MD, USA), supplemented with 10% fetal bovine serum (FBS500-Y; HYCEZMBIO, Wuhan, China), 1% penicillin-streptomycin solution, and 1% sodium pyruvate (11360070; Gibco, Baltimore, MD, USA), at 37 °C in a humidified incubator containing 5% CO_2_.

Transient transfections were performed using NEOFECT™ DNA transfection reagent (TF201201; Neofect, Beijing, China) according to the manufacturer’s instructions. Cells were harvested for downstream assays 48 h after transfection unless otherwise specified.

### 2.3. Plasmid Construction and RNA Oligonucleotides

The previously obtained *ERVWE1* sequence [40] was cloned into the mammalian expression vector pcDNA 3.1 vector in our laboratory. Plasmids pCMV-BNIP3-3*FLAG-NEO (P51135; Miaoling, Wuhan, China) and pCMV-3*FLAG (P1303; Miaoling, Wuhan, China) were obtained commercially. Short hairpin RNAs (shRNAs) targeting BNIP3 and a negative control (shNC) were cloned into the pSilencer 2.1-U6 neo shRNA expression vector using *Bam*HI-HF (R3136S; New England Biolabs, Ipswich, MA, USA) and *Hind*III (R3104S; New England Biolabs, Ipswich, MA, USA) restriction enzymes.

MiR-27b-3p mimic and corresponding negative controls were purchased from Sangon Biotech (Shanghai, China). All plasmids and oligonucleotides were sequence-verified by Sangon Biotech prior to use.

### 2.4. Bioinformatic Analysis

Gene expression profiles from the hippocampus and prefrontal cortex of schizophrenia patients and matched controls were retrieved from the GEO database (accession number: GSE53987). Differentially expressed genes (DEGs) were visualized using volcano plots and heat maps generated with the SangerBox online platform. Gene Ontology (GO) biological process and KEGG pathway enrichment analyses were performed using the same platform, with statistical significance defined as *p* < 0.05.

### 2.5. ELISA

Plasma concentrations of ERVWE1 (LB111222B; Liberi Bio, Wuhan, China) and BNIP3 (MM-51677H1; Enzyme Immunity, Yancheng, China) were quantified using commercially available ELISA kits according to the manufacturers’ protocol. Absorbance was measured at 450 nm using a spectrophotometer. Protein concentrations were calculated based on standard curves generated for each assay.

### 2.6. Immunofluorescence and Confocal Microscopy

SH-SY5Y cells were seeded onto confocal dishes and subjected to the indicated treatments. Forty-eight hours after transfection, cells were stained with MitoTracker Red CMXRos (IF1770; Solarbio, Beijing, China) for 30 min at 37 °C, followed by nuclear counterstaining with Hoechst (C0030; Solarbio, Beijing, China) dye. The opening of mitochondria permeability transition pores (MPTPs) were investigated with MPTPs Assay Kit (C2009S; Beyotime, Shanghai, China) respectively according to the manufactures’ instructions. Images were captured using a confocal laser microscope (TCS SP8; Leica Microsystems, Wetzlar, Germany) equipped with an HCX PL APO 63x/1.40 oil objective. Images were processed and analyzed in two dimensions using green (excitation: 488 nm), red (excitation: 552 nm), and blue (excitation: 405 nm) channels. Mitochondrial morphology was quantified using ImageJ (v1.8.0) software. All experiments were independently repeated at least three times.

### 2.7. Quantitative Real-Time PCR (qRT-PCR)

Total RNA was extracted using TRIzol reagent (15596018; Invitrogen, Carlsbad, CA, USA). Reverse transcription was performed using 1 μg of total RNA (FSK-101; TOYOBO, Osaka, Japan). Quantitative PCR was carried out using SYBR Select Master Mix (2992239AX; Aidlab Biotechnologies Co. Ltd., Beijing, China) on a real-time PCR system. Glyceraldehyde-3-phosphate dehydrogenase (GAPDH) was used as the internal control for mRNA normalization. Relative expression levels were calculated using the 2^−ΔΔCt^ method. Primer sequences were listed in Supplementary Table S2.

### 2.8. Western Blot

Cells were lysed in M-PER™ Mammalian Protein Extraction Reagent (UC282138; Thermo Fisher Scientific, Waltham, MA, USA) supplemented with protease and phosphatase inhibitors (P8340; Sigma, Steinheim, Germany). Protein concentration was determined using a BCA Protein Assay Kit (23250; Thermo Fisher Scientific, Waltham, MA, USA). Equal amounts of protein were separated by 12% SDS-PAGE gels and transferred onto PVDF membranes (ISEQ00010; Millipore, Burlington, MA, USA). Primary antibody species were listed in Supplementary Table S3. Membranes were incubated with primary antibodies overnight at 4 °C, followed by incubation with secondary antibodies goat anti-mouse IgG-HRP (1:10,000; AS003; Abclonal, Wuhan, China) or goat anti-rabbit IgG-HRP (1:10,000; AS014; Abclonal, Wuhan, China). Protein bands were visualized using ECL chemiluminescence reagent (SW2030; Biosharp, Hefei, China) via an automated chemiluminescence system (5200; Tanon, Shanghai, China) and quantified using ImageJ (v1.8.0) software.

### 2.9. Dual-Luciferase Assay

The predicted miR-27b-3p binding site within the *BNIP3* 3′ untranslated region (3′UTR) was cloned into the pMIR-GLO dual-luciferase miRNA target expression vector. Luciferase activity was measured using the Dual-Luciferase Reporter Assay Kit (DL101; Vazyme, Nanjing, China) according to the manufacturer’s protocol, and firefly luciferase activity was normalized to Renilla luciferase activity.

### 2.10. Statistical Analysis

Statistical analyses were performed using SPSS 20 and GraphPad Prism 8.0. Clinical data were analyzed using non-parametric tests and are presented as medians. Correlation analysis was conducted using Spearman’s rank correlation. Experimental data are presented as mean ± standard deviation (SD). Comparisons between groups were performed using Student’s *t*-test or one-way ANOVA as appropriate. A *p*-value < 0.05 was considered statistically significant. All experiments were repeated at least three times.

## 3. Results

### 3.1. BNIP3 Expression Is Elevated in Schizophrenia and Positively Correlates with ERVWE1

**Figure 1:**
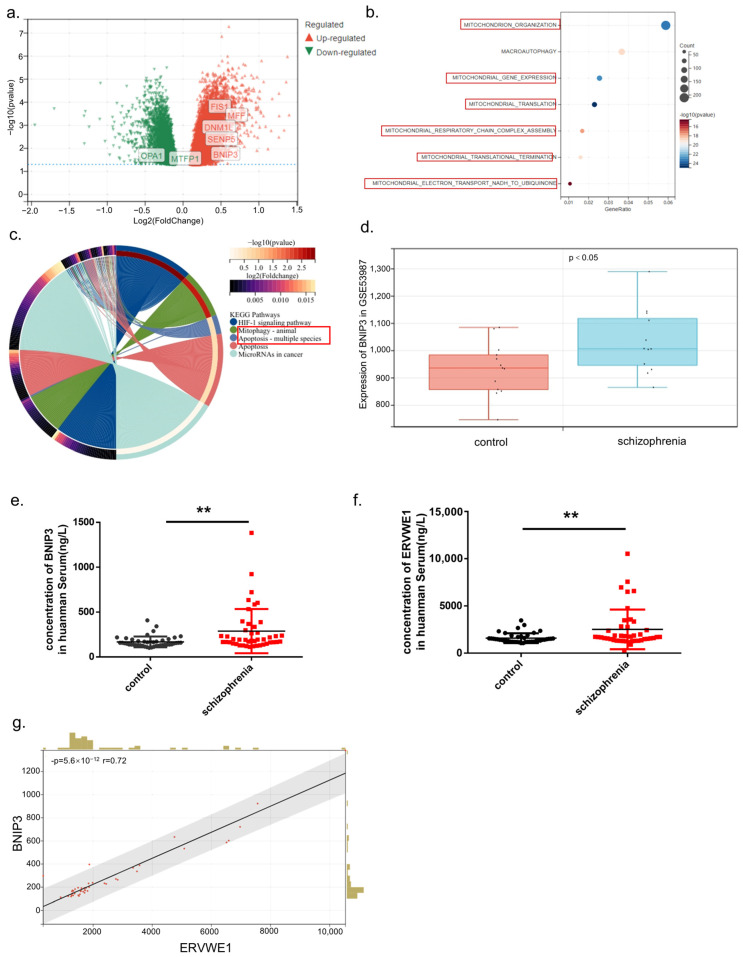
BNIP3 is upregulated in Schizophrenia and positively Correlates with ERVWE1. (**a**) Volcano plot and expression profiling of mitochondria-related differentially expressed genes (DEGs) identified from dataset GSE53987. (**b**) GOBP enrichment analysis of DEGs from GSE53987 (X-axis, gene ratio; Y-axis, GO terms circle size shows gene count). The red boxes indicate processes related to mitochondrial biogenesis. (**c**) KEGG pathway enrichment analysis of DEGs from GSE53987. Red boxes indicate apoptotic pathways. (**d**) *BNIP3* mRNA expression levels in schizophrenia patients compared with healthy controls in GSE53987. (**e**,**f**) Plasma concentration of ERVWE1 and BNIP3 in schizophrenia patients and healthy controls. (**g**) Pearson correlation analysis showing a significant positive correlation between ERVWE1 protein and BNIP3 protein levels in patient plasma, r = 0.72. ** *p* < 0.01.

Given the well-established association between mitochondrial dysfunction and schizophrenia pathology [11], we first sought to identify mitochondrial-related genes that may link viral elements to mitochondrial impairment in schizophrenia. To this end, we performed bioinformatic analysis using the public transcriptomic dataset GSE53987, which includes gene expression profiles from multiple brain regions—namely prefrontal cortex, hippocampus, striatum-of schizophrenia patients and matched healthy controls [49]. Differential expression analysis revealed that numerous mitochondria-related genes were significantly dysregulated in the brain tissues of SCZ patients, among which *BNIP3* was prominently upregulated (Figure 1a). Gene ontology biological process (GOBP) and KEGG pathway enrichment analyses of differentially expressed genes in the hippocampus further demonstrated significant enrichment of mitochondria dysfunction-related and apoptosis-related pathways (Figure 1b,c). Consistent with these findings, *BNIP3* mRNA expression was significantly elevated in schizophrenia patients compared with controls across multiple brain regions in the GSE53987 dataset (Figure 1d), supporting the notion that mitochondrial dysfunction is a core feature of schizophrenia [12].

To validate these findings at the protein level and explore their clinical relevance, we next examined BNIP3 expression in peripheral blood samples from schizophrenia patients. There were no significant differences in age, level of education, body mass index (BMI), gender distribution, nor smoking status between schizophrenic patients and healthy controls (Supplementary Table S1). ELISA analysis revealed a significant increase in BNIP3 protein levels in the blood of schizophrenia patients compared with healthy controls (Figure 1e,f). Importantly, correlation analysis demonstrated a significant positive correlation between BNIP3 protein levels and ERVWE1 protein expression in patient samples (Figure 1g). Given the established role of BNIP3 in mitochondrial quality control, mitophagy and apoptosis [23,24], these data suggest a potential functional link between ERVWE1 and BNIP3 in the mitochondrial pathology of schizophrenia.

### 3.2. ERVWE1 Induces Mitochondrial Damage Through Upregulation of BNIP3

**Figure 2:**
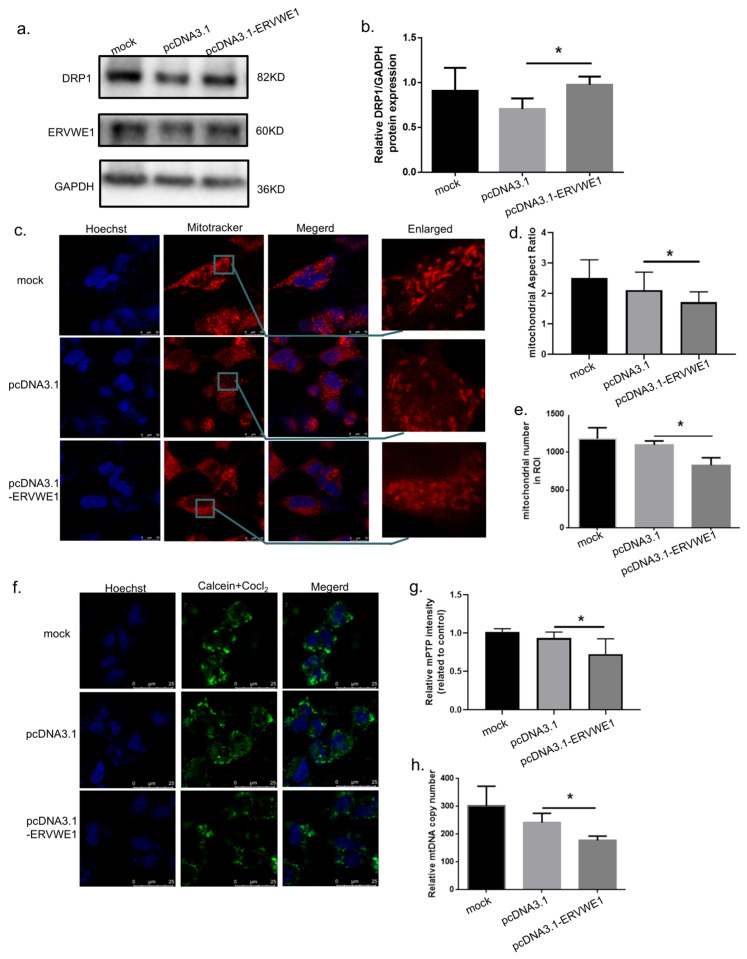
ERVWE1 induces Mitochondrial Structural Damage. (**a**,**b**) Western blot analysis and quantification of DRP1 protein levels. (**c**–**e**) Representative images (red: Mitotracker; blue: Hoechst) and quantitative analysis of mitochondrial morphology following ERVWE1 overexpression, including mitochondrial aspect ratio and mitochondrial number. (**f**,**g**) Assessment of mPTP opening after ERVWE1 overexpression using Calcein-AM staining (green: Calcein-AM; blue: Hoechst); and corresponding quantitative analysis. (**h**) qPCR analysis of mitochondrial DNA copy number (mtDNA CN). Statistical analysis: one-way ANOVA and Student’s *t*-test. * *p* < 0.05.

Previous studies have reported that ERVWE1 induces mitochondrial fragmentation [50]. We therefore investigated whether ERVWE1 also causes broader mitochondrial structural and functional alterations. Successful expression was confirmed by RT-qPCR and Western blotting analysis (Supplementary Figure S1). In SH-SY5Y cells overexpressing ERVWE1, we observed an abnormal elevation of DRP1, a mitochondrial fission protein (Figure 2a,b). Using Mitotracker staining and quantitative mitochondrial morphology analysis, we observed that ERVWE1 overexpression in SH-SY5Y cells resulted in marked mitochondrial abnormalities, including a significant reduction in mitochondrial aspect ratio and mitochondrial number (Figure 2c–e). In parallel, Calcein AM staining revealed a pronounced increase in mitochondrial permeability transition pore (mPTP) opening following ERVWE1 overexpression (Figure 2f,g). Furthermore, qPCR analysis demonstrated a significant decrease in mitochondrial DNA copy number (mtDNA CN) (Figure 2h), indicating impaired mitochondrial integrity.

**Figure 3:**
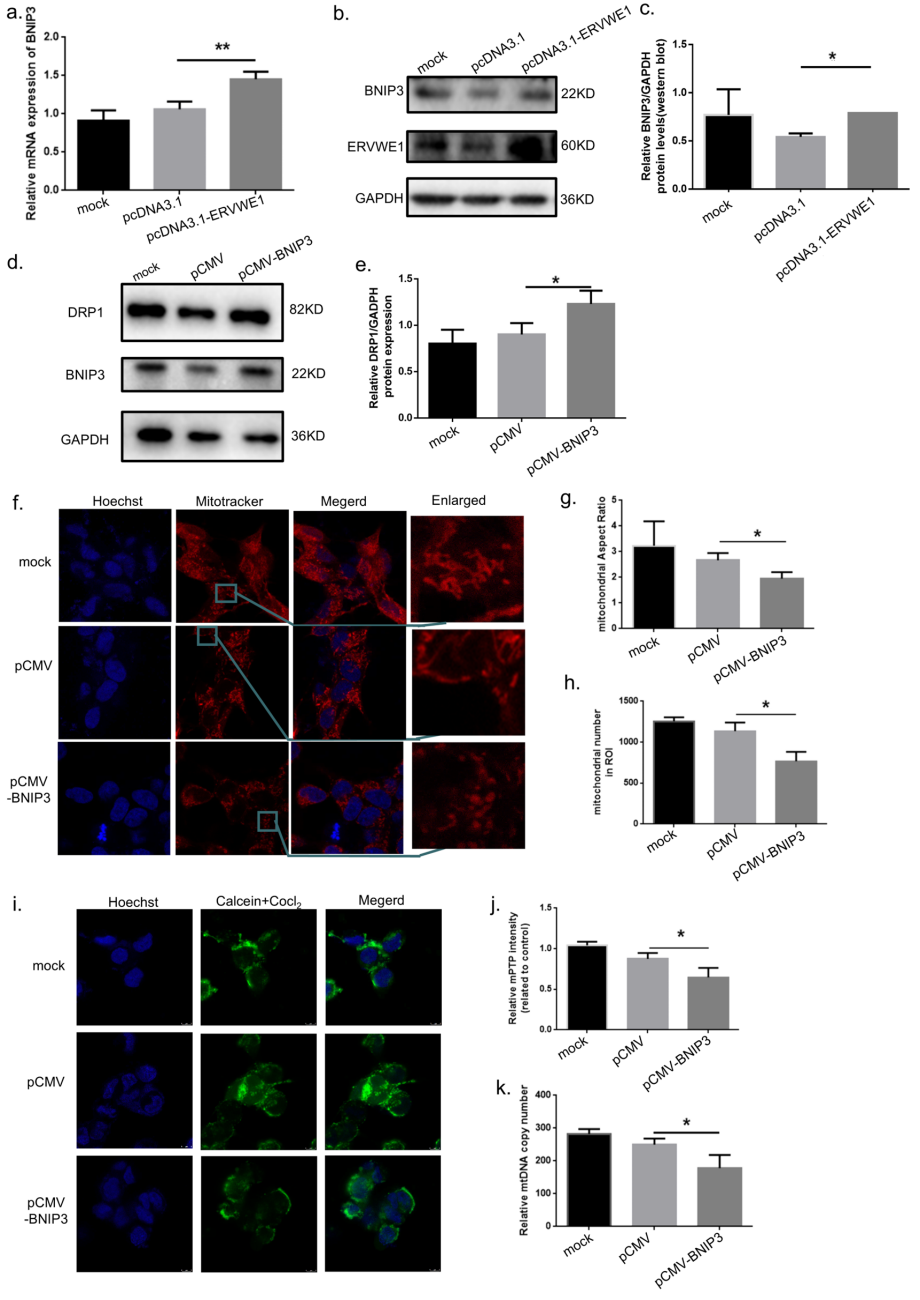
ERVWE1 upregulates BNIP3 expression and BNIP3 overexpression recapitulates ERVWE1-induced mitochondrial damage. (**a**) qPCR analysis of *BNIP3* mRNA levels following ERVWE1 overexpression. (**b**,**c**) Western blot analysis and quantification of BNIP3 protein levels. (**d**,**e**) Western blot analysis and quantification of DRP1 protein levels. (**f**–**h**) Representative images (red: Mitotracker; blue: Hoechst) and quantitative analysis of mitochondrial morphology following BNIP3 overexpression, including mitochondrial aspect ratio and mitochondrial number. (**i**,**j**) Detection and quantification of mPTP opening following BNIP3 overexpression using Calcein-AM staining (green: Calcein-AM; blue: Hoechst). (**k**) qPCR analysis of mtDNA CN. Statistical analysis: one-way ANOVA and Student’s *t*-test. * *p* < 0.05, ** *p* < 0.01.

Given the observed correlation between ERVWE1 and BNIP3 in clinical samples, we next examined whether ERVWE1 regulates BNIP3 expression at the cellular level. Overexpression of ERVWE1 (pcDNA3.1-ERVWE1) in SH-SY5Y cells significantly increased both BNIP3 mRNA and protein levels, as confirmed by qPCR and Western Blot analyses (Figure 3a–c). To determine whether BNIP3 is sufficient to induce mitochondrial damage, we overexpressed BNIP3 (pCMV-BNIP3) in SH-SY5Y cells. Efficient expression of BNIP3 was confirmed at the mRNA and protein level (Supplementary Figure S2). BNIP3 overexpression recapitulated the mitochondrial phenotypes induced by ERVWE1, including an abnormal elevation of DRP1 (Figure 3d,e), reduced mitochondrial aspect ratio (Figure 3f–h), enhanced mPTP opening (Figure 3i,j), and decreased mtDNA CN (Figure 3k).

**Figure 4:**
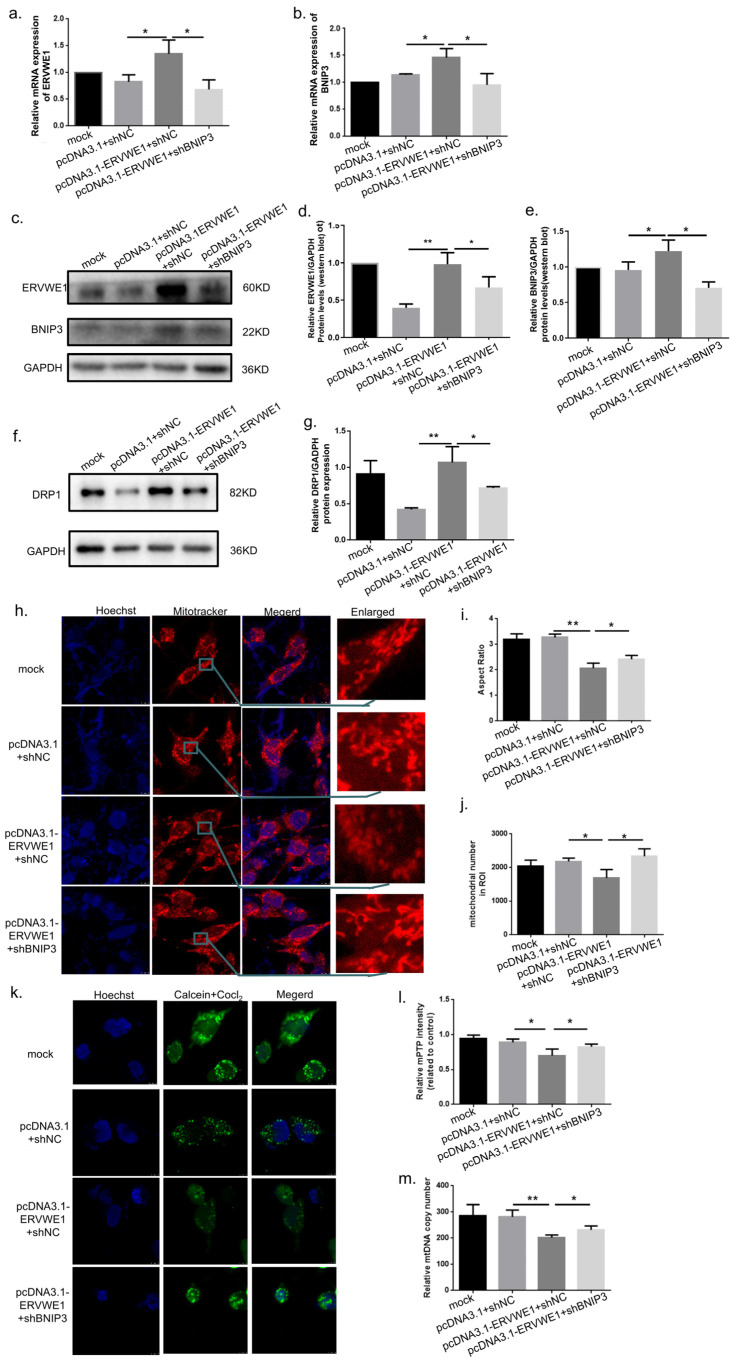
BNIP3 knockdown reverses ERVWE1-induced mitochondrial damage. (**a**,**b**) qPCR analysis of *BNIP3* and *ERVWE1* mRNA expression following BNIP3 knockdown (shBNIP3) in the presence of ERVWE1 overexpression. (**c**–**e**) Western blot analysis and quantification of BNIP3 and ERVWE1 protein levels. (**f**,**g**) Western blot analysis and quantification of DRP1 protein levels. (**h**–**j**) Representative images (red: Mitotracker; blue: Hoechst) and quantitative analysis of mitochondrial morphology following shBNIP3 treatment combined with ERVWE1 overexpression, including mitochondrial aspect ratio and mitochondrial number. (**k**,**l**) Assessment and quantification of mPTP opening using Calcein-AM staining (green: Calcein-AM; blue: Hoechst). (**m**) qPCR analysis of mtDNA CN. Statistical analysis: one-way ANOVA and Student’s *t*-test. * *p* < 0.05, ** *p* < 0.01.

To directly test whether BNIP3 mediates ERVWE1-induced mitochondrial damage, we performed rescue experiments using a BNIP3-specific short hairpin RNA (shBNIP3). Successful knockdown of BNIP3 was confirmed (Supplementary Figure S3). While ERVWE1 overexpression robustly increased BNIP3 mRNA and protein levels (Figure 4a–e), co-transfection with shBNIP3 significantly rescued ERVWE1-induced abnormal elevations of DRP1 (Figure 4f,g), mitochondrial morphological abnormalities (Figure 4h–j), excessive mPTP opening (Figure 4k,l), and in mtDNA CN reduction (Figure 4m). These results demonstrate that BNIP3 functions as a critical downstream effector molecule of ERVWE1 in mediating mitochondrial structural and functional damage.

### 3.3. ERVWE1 Regulates BNIP3 Expression Through Suppression of miR-27b-3p

**Figure 5:**
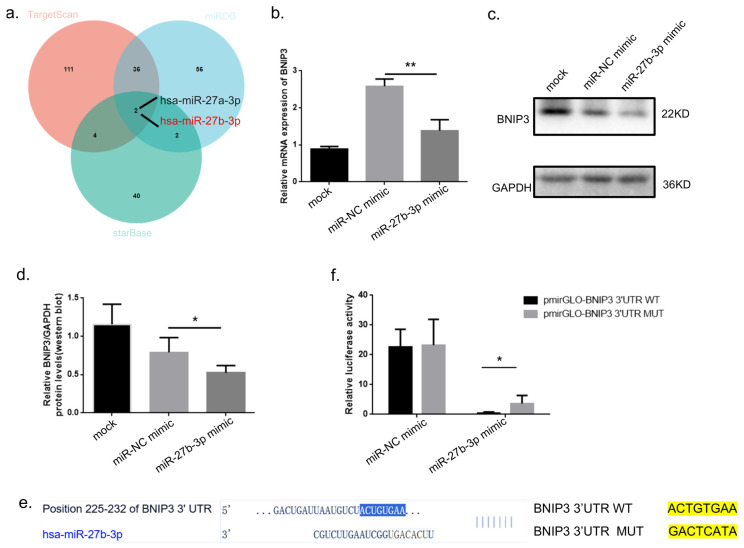
miR-27b-3p Directly Targets and Negatively Regulates BNIP3 Expression. (**a**) Bioinformatic prediction of miRNAs targeting BNIP3 and identification of common candidates using three independent databases. (**b**) qPCR analysis of *BNIP3* mRNA expression following transfection with miR-27b-3p mimic. (**c**,**d**) Western blot analysis and quantification of BNIP3 protein levels after miR-27b-3p mimic transfection. (**e**) Predicted binding site between miR-27b-3p and the *BNIP3* 3′UTR, including wild-type and mutant sequences. (**f**) Dual-luciferase reporter assay validating the direct interaction between miR-27b-3p and the *BNIP3* 3′UTR. Statistical analysis: one-way ANOVA. * *p* < 0.05, ** *p* < 0.01.

Having established BNIP3 as a key mediator of ERVWE1-induced mitochondrial dysfunction, we next sought to elucidate the molecular mechanism regulating BNIP3 expression. Bioinformatic analysis identified BNIP3 as a putative target of miR-27b-3p (Figure 5a). Efficient expression of miR-27b-3p mimic in SH-SY5Y cells was confirmed (Supplementary Figure S4a). Functional validation demonstrated that transfection with a miR-27b-3p mimic significantly reduced endogenous BNIP3 protein levels (Figure 5b–d). To confirm the specificity of this interaction, the predicted miR-27b-3p binding site within the *BNIP3* 3′UTR was mutated (Figure 5e). Dual-luciferase reporter assays further revealed that the miR-27b-3p mimic markedly suppressed the luciferase activity of the wild-type *BNIP3* 3′UTR reporter, whereas no significant effect was observed for the mutant construct (Figure 5f). Together, these results provide direct and robust evidence that BNIP3 is a bona fide target of miR-27b-3p.

**Figure 6:**
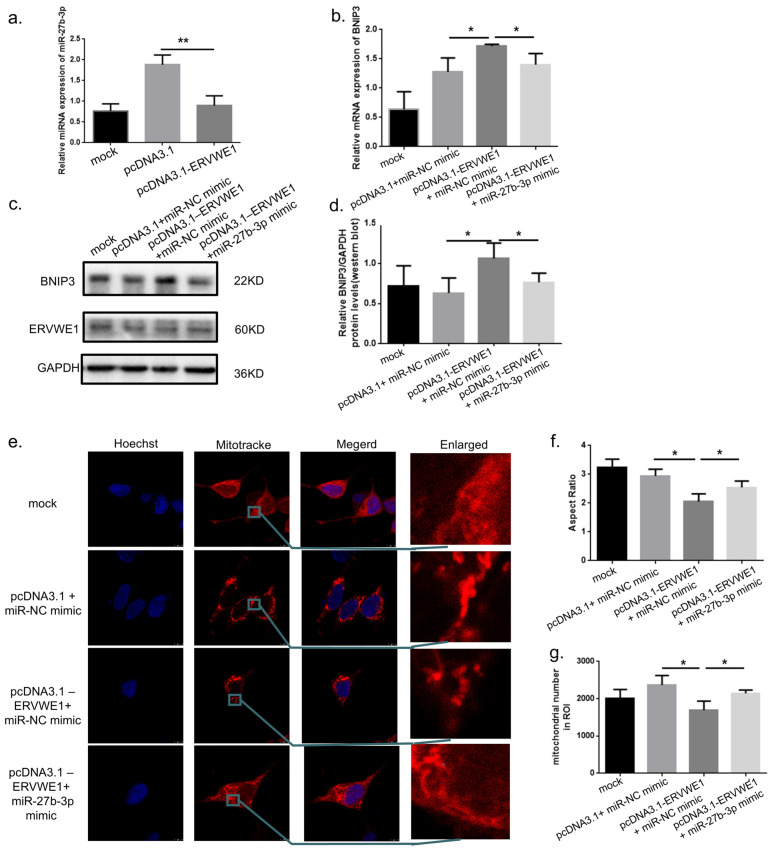

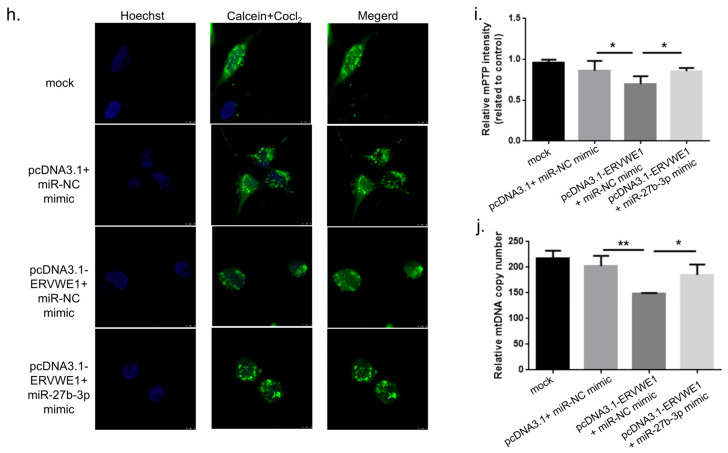
Involvement of the miR-27b-3p/BNIP3 signaling pathway in ERVWE1-induced Mitochondrial Damage. (**a**,**b**) qPCR analysis of *BNIP3* and *ERVWE1* mRNA expression following miR-27b-3p mimic transfection with ERVWE1 overexpression. (**c**,**d**) Western blot analysis and quantification of BNIP3 and ERVWE1 protein levels. (**e**–**g**) Representative images (red: Mitotracker; blue: Hoechst) and quantitative analysis of mitochondrial morphology following miR-27b-3p mimic transfection combine with ERVWE1 overexpression, including mitochondrial aspect ratio and mitochondrial number. (**h**,**i**) Detection and quantification of mPTP opening using Calcein-AM staining (green: Calcein-AM; blue: Hoechst). (**j**) qPCR analysis of mtDNA CN. Statistical analysis: one-way ANOVA and Student’s *t*-test. * *p* < 0.05, ** *p* < 0.01.

Given the established role of miRNAs in neuropsychiatric disorders [35,36], we next examined whether ERVWE1 regulates BNIP3 expression through miR-27b-3p. qPCR analysis revealed that ERVWE1 overexpression significantly downregulated miR-27b-3p expression (Figure 6a). The transfection efficiency of ERVWE1 and miR-27b-3p expression in SH-SY5Y cells co-transfected with ERVWE1 and miR-27b-3p mimic were confirmed (Supplementary Figure S4b,c). Importantly, co-transfection of a miR-27b-3p mimic in ERVWE1-overexpressing cells effectively reversed BNIP3 upregulation at both the mRNA and protein levels (Figure 6b–d). Moreover, restoration of miR-27b-3p significantly ameliorated ERVWE1-induced mitochondrial abnormalities, including mitochondrial morphological defects (Figure 6e–g), excessive mPTP opening (Figure 6h,i) and mtDNA CN reduction (Figure 6j). These findings establish a regulatory ERVWE1/miR-27b-3p/BNIP3 signaling axis governing mitochondrial integrity.

### 3.4. ERVWE1-Induced Mitochondrial Damage Promotes Cytochrome c Release and Neuronal Apoptosis via BNIP3

**Figure 7:**
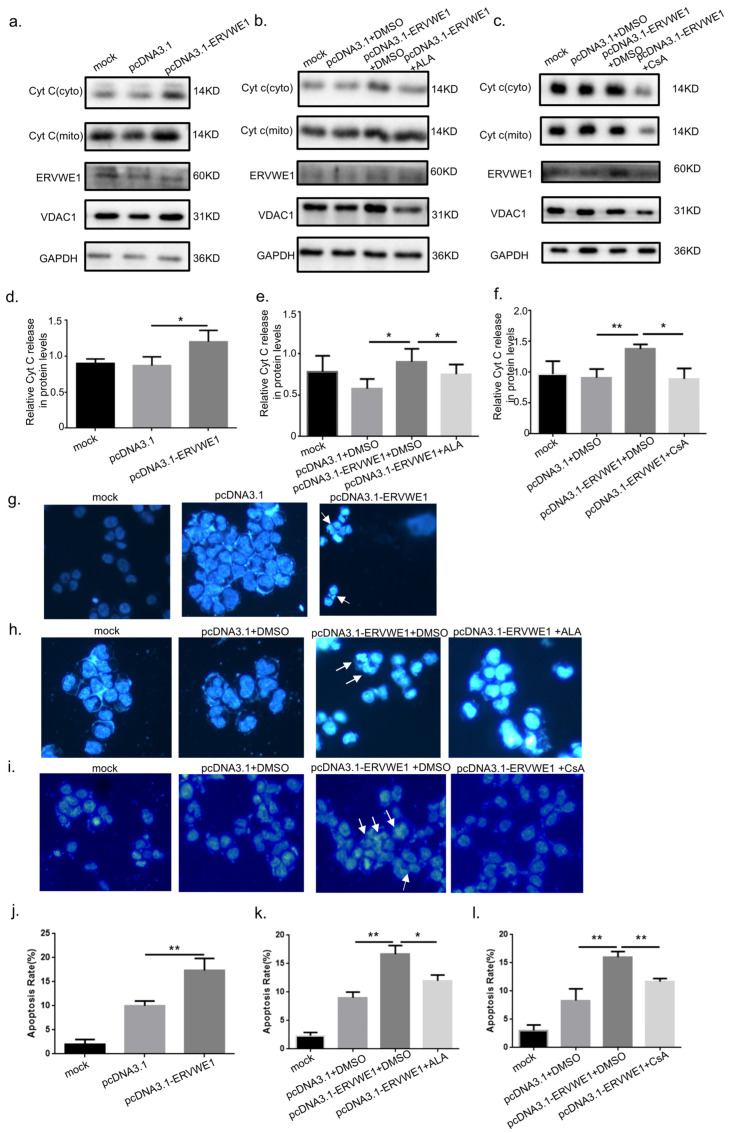
ERVWE1 Leads to Cytochrome c Release and Induces Apoptosis. (**a**–**f**) Western Blot analysis of cytosolic and mitochondrial fractions in SH-SY5Y cells, showing ERVWE1-induced cytochrome c (Cyt c) release. (**g**–**l**) Representative images (blue: Hoechst; arrows indicate apoptotic nuclei) and quantitative analysis of apoptotic cells and apoptosis rate. Statistical analysis: one-way ANOVA and Student’s *t*-test. * *p* < 0.05, ** *p* < 0.01.

Mitochondrial structural damage and mPTP opening are known to trigger cytochrome c release, a critical event in the intrinsic apoptotic pathway. We therefore examined whether ERVWE1-induced mitochondrial dysfunction leads to apoptosis. Subcellular fractionation followed by Western Blot analysis revealed that ERVWE1 overexpression significantly decreased cytochrome c (Cyt c) in the mitochondria fraction while increasing its accumulation in the cytosolic fraction, indicating cytochrome c release from mitochondria into the cytosol (Figure 7a,d). Subsequently, released cytochrome c leads to apoptosis (Figure 7g,j). Pharmacological interventions further confirmed the mitochondrial origin of apoptosis. Co-treatment with α-lipoic acid (ALA), a compound known to protect mtDNA integrity, significantly attenuated ERVWE1-induced cytochrome c release (Figure 7b,e) and reduced apoptosis (Figure 7h,k). Similarly, Cyclosporin A (CsA), an inhibitor of mPTP opening, markedly suppressed cytochrome c release and apoptosis in ERVWE1-overexpressing cells (Figure 7c,f,i,l). These results indicate that mtDNA damage and mPTP opening are key upstream events linking ERVWE1-induced mitochondrial dysfunction to apoptotic execution.

**Figure 8:**
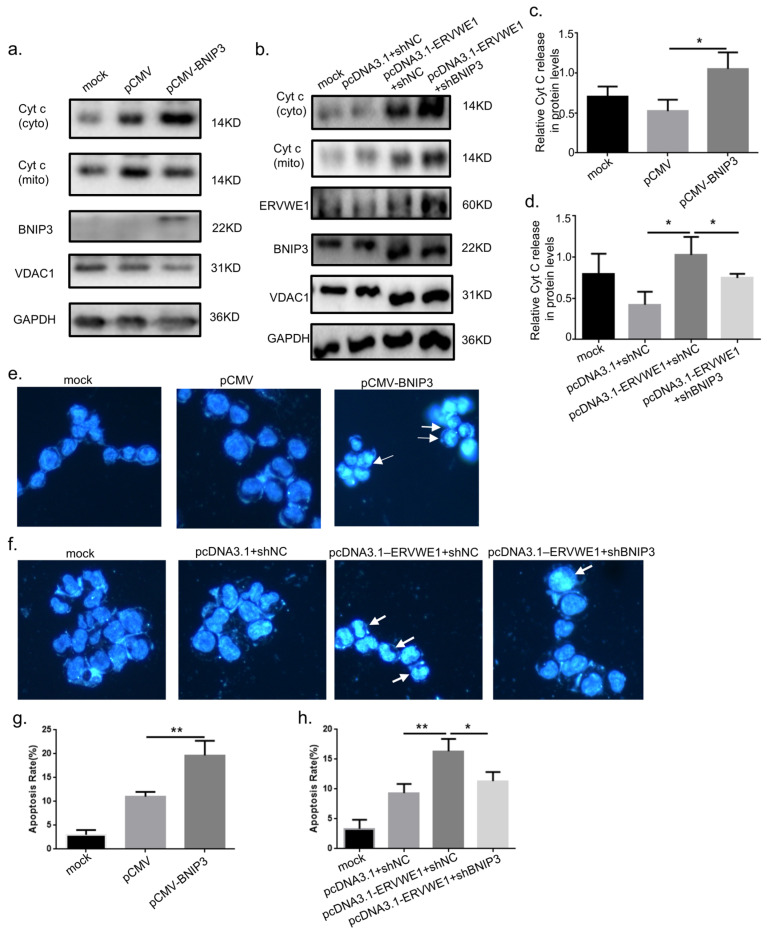
BNIP3 mediates ERVWE1-induced mitochondrial apoptosis via cytochrome c release. (**a**–**d**) Western blot analysis and quantification of cytochrome c release in protein levels. (**e**–**h**) Representative images (blue: Hoechst; arrows indicate apoptotic nuclei) and quantitative analysis of apoptosis rate. Statistical analysis: one-way ANOVA and Student’s *t*-test. * *p* < 0.05, ** *p* < 0.01.

To determine the specific role of BNIP3 in ERVWE1-mediated apoptosis, we manipulated BNIP3 expression directly. Overexpression of BNIP3 (pCMV-BNIP3) alone significantly increased cytochrome c release and the final apoptosis rate (Figure 8a,c,e,g). Strikingly, these effects were almost completely abolished by co-transfection with shBNIP3, whereas a negative control shRNA had no protective effect (Figure 8b,d,f,h). Together, these findings demonstrate that BNIP3 is both necessary and sufficient for ERVWE1-induced mitochondrial apoptosis, functioning by promoting cytochrome c release from damaged mitochondria and activating the downstream apoptotic cascade in neuronal cells.

## 4. Discussion

**Figure 9:**
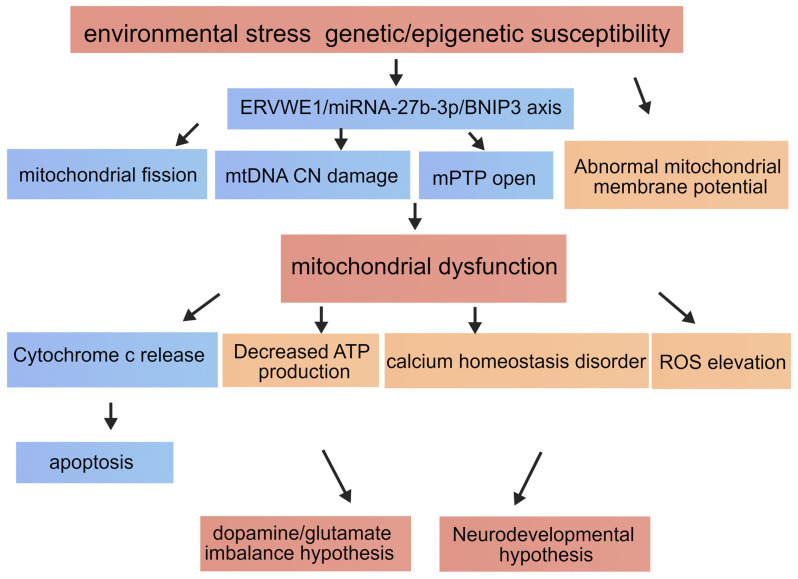
Integration of the ERVWE1/miR-27b-3p/BNIP3 Axis into the Mitochondrial Dysfunction Network of Schizophrenia. The ERVWE1/miR-27b-3p/BNIP3 axis and its effects are highlighted in blue boxes. Other mechanisms and hypotheses are highlighted in red and orange boxes. Arrows indicate the direction of regulatory relationships and the cascade of pathological events.

Schizophrenia is a highly heterogeneous neuropsychiatric disorder with multifactorial etiologies involving genetic susceptibility, environmental exposures, and disrupted neurodevelopment. Accumulating evidence indicates that mitochondrial dysfunction represents a central pathological hub through which diverse risk factors converge (Figure 9). In the present study, we identify a previously unrecognized molecular path-way—ERVWE1/miR-27b-3p/BNIP3 axis—in which ERVWE1 promotes BNIP3 upregulation by suppressing miR-27b-3p, while BNIP3 is directly targeted by miR-27b-3p. By integrating bioinformatic analyses, clinical sample validation, and mechanistic cellular experiments, our findings provide a coherent framework connecting viral elements to mitochondrial pathology and neuronal fate.

Previous studies from our laboratory and others have consistently shown that ERVWE1 is upregulated in schizophrenia and contributes to disease pathology through multiple independent yet interconnected mechanisms. ERVWE1 has been reported to regulate the expression of schizophrenia-associated genes, including brain-derived neurotrophic factor (*BDNF*) [51] and disrupted in schizophrenia 1 (*DISC1*) [52]. It also modulates neuronal ion channel function, such as small conductance Ca^2+^-activated K^+^ channel type II (SK2) [53], SK3 [54] and sodium channels [55]. Beyond gene regulation and electrophysiological modulation, ERVWE1 induces organelle dysfunction by triggering endoplasmic reticulum stress [56], promoting mitochondrial fragmentation [50], and impairing mitochondrial respiratory chain activity [57]. It further disrupts neuronal morphology and synaptic architecture through Wnt/JNK signaling pathway [58] and epitranscriptomic regulation [59]. Functionally, ERVWE1 has been shown to induce multiple forms of programmed neuronal cell death, including apoptosis [60], ferroptosis [61], and pyroptosis [44]. In parallel, ERVWE1 exerts potent immunomodulatory effects, activating both innate and adaptive immune responses within the central nervous system [60,62,63,64,65]. Despite this extensive body of evidence, a unifying mechanism that links ERVWE1 activation to core intracellular pathological processes in schizophrenia has remained elusive. Our study addresses this gap by identifying mitochondrial dysfunction as a critical downstream consequence of ERVWE1 activation and by positioning BNIP3 as a key molecular effector mediating ERVWE1-driven mitochondrial pathology.

Using the widely adopted GSE53987 transcriptomic dataset [66], we confirmed broad dysregulation of mitochondria-related genes in schizophrenia, particularly in the hippocampus—a brain region critically involved in cognition and emotional regulation. Among these genes, *BNIP3* emerged as a consistently upregulated mitochondrial protein. This observation extends prior findings that mitochondrial genes such as *ATP5D* are altered across multiple brain regions in schizophrenia [67] and reinforces the hypothesis that mitochondrial dysfunction is a core pathological feature of the disorder [68]. Importantly, we validated BNIP3 upregulation at the protein level in the peripheral blood of schizophrenia patients and demonstrated a significant positive correlation between BNIP3 and ERVWE1 expression. This dual validation bridges bioinformatic predictions with clinical observations and supports the notion that peripheral BNIP3 levels may serve as a biologically plausible and peripherally detectable candidate indicator of cellular stress associated with ERVWE1 activation in schizophrenia, pending further direct validation of its relationship to central nervous system pathology.

Mounting evidence implicates mitochondrial dysfunction as a core pathological feature of schizophrenia [68]. Building on our previous finding that the schizophrenia-associated risk factor ERVWE1 impairs ATP synthesis and mitochondrial membrane potential (MMP) [50], we now provide a novel molecular explanation for this effect. Specifically, ERVWE1 activation induces profound mitochondrial damage, as evidenced by decreased mitochondrial aspect ratio, and a reduction in mtDNA copy number. These findings directly link ERVWE1 activation to ultrastructural and functional mitochondrial defects, offering a new etiological perspective on schizophrenia. BNIP3 is a mitochondrial outer membrane protein that plays a pivotal role in mitophagy and mitochondrial quality control [24]. While its physiological activation promotes the clearance of damaged mitochondria, excessive or sustained BNIP3 expression can drive pathological outcomes, including mPTP opening, mitochondrial membrane potential collapse, and apoptosis [69]. This dual-edged nature of BNIP3 has been reported in neurodegenerative disorders [70] such as Parkinson’s disease, where controlled BNIP3/NIX activation is neuroprotective, whereas dysregulation leads to neuronal injury [71]. Although BNIP3 has been shown to induce mPTP opening [25], its effects on mtDNA copy number have not previously been characterized. Our results reveal that in SH-SY5Y cells, BNIP3 not only triggers mPTP opening but also leads to a significant reduction in mtDNA copy number. Importantly, our data extend these observations to schizophrenia, demonstrating for the first time that BNIP3 overexpression alone is sufficient to reproduce the mitochondrial damage phenotype induced by ERVWE1. Conversely, BNIP3 knockdown effectively rescues ERVWE1-mediated mitochondrial abnormalities. Together, these findings identify BNIP3 as an indispensable downstream effector linking ERVWE1 activation to mitochondrial dysfunction and establish a mechanistic axis through which ERVWE1 contributes to schizophrenia-associated mitochondrial pathology.

MicroRNA dysregulation is a well-established feature of schizophrenia and plays a critical role in shaping neuronal gene regulatory networks [72]. BNIP3 is known to be regulated at both transcriptional and post-transcriptional levels, suggesting that it functions as a finely tuned molecular node susceptible to dysregulation in disease states [28,29]. Notably, the regulatory role of miR-27b-3p extends beyond schizophrenia and appears to have broader pathophysiological relevance, acting as a context-dependent regulator in multiple neuro-degenerative disorders. In Alzheimer’s disease (AD), circulating levels of miR-27b-3p have been shown to correlate significantly with amyloid burden, suggesting a potential involvement in disease progression [73]; Similarly, in Parkinson’s disease (PD), miR-27b-3p expression is significantly elevated in peripheral blood mononuclear cells, particularly during the early stages of the disease, indicating its association with early neurodegenerative processes [74]. In this study, we identify miR-27b-3p as a direct post-transcriptional regulator of BNIP3 and demonstrate that ERVWE1 suppresses miR-27b-3p expression, thereby relieving repression of BNIP3. Through dual-luciferase reporter assays and functional rescue experiments, we provide definitive evidence that miR-27b-3p directly targets the *BNIP3* 3′UTR and that restoration of miR-27b-3p expression effectively reverses ERVWE1-induced BNIP3 upregulation and mitochondrial damage. This finding mechanistically links viral activation to miRNA dysregulation and mitochondrial pathology, bridging molecular events across multiple regulatory layers. In the context of emerging network medicine and single-cell transcriptomic approaches in schizophrenia, our work adds a concrete virus–miRNA–mitochondrial protein axis that may serve as a tractable target for future therapeutic development.

Mitochondrial structural damage and mPTP opening represent critical checkpoints in the initiation of intrinsic apoptosis [20]. Although increased neuronal apoptosis has been observed in schizophrenia [60], the upstream molecular triggers initiating this cascade have remain unclear. Our study demonstrates that ERVWE1-induced mitochondrial damage via BNIP3 directly promotes cytochrome c release into the cytosol, activating the intrinsic apoptotic pathway. This provides a mechanistic explanation linking mitochondrial dysfunction to neuronal loss in schizophrenia. Notably, we show that pharmacological interventions targeting mitochondrial integrity—such as α-lipoic acid, which protects mtDNA, and cyclosporin A, an mPTP inhibitor [75]—can effectively block cytochrome c release and apoptosis. These findings not only reinforce the central role of mitochondria in schizophrenia pathology but also provide experimental support for mitochondria-targeted therapeutic strategies. Moreover, this pathway offers a mechanistic explanation for clinical observations such as elevated mitochondrial-derived circulating cell-free DNA in schizophrenia [76], suggesting that targeting mitochondrial apoptosis may have dual benefits for both treatment and biomarker development.

Our study identifies, for the first time, the involvement of the ERVWE1/miR-27b-3p/BNIP3 axis in the adult pathology of schizophrenia. Notably, all three components of this axis play established roles in placental biology: ERVWE1 (Syncytin-1) is essential for syncytio-trophoblast formation [77] and the establishment of immune tolerance at the maternal–fetal interface [78]. MiR-27b-3p participates in the regulation of placental angiogenesis and trophoblast differentiation [79], while BNIP3 mediates mitophagy in response to placental hypoxia and cellular stress [80]. Based on these observations, we speculate that genetic susceptibility or environmental stressors during pregnancy may lead to aberrant ERVWE1 expression in the placenta. Resultant placental oxidative or inflammatory stress could disrupt miR-27b-3p homeostasis, thereby promoting BNIP3 upregulation. Such dysregulation may expose the developing fetus to a chronic stressful intrauterine environment, potentially “programming” mitochondrial function and cellular stress response pathways in the developing brain. This developmental priming could increase vulnerability to later-life activation of the same ERVWE1/miR-27b-3p/BNIP3 axis, contributing to schizophrenia pathology in adulthood. Future studies employing developmental model systems and prospective clinical cohorts will be required to directly test this hypothesis and to clarify the temporal and causal relationships underlying this proposed mechanism.

Schizophrenia arises from a combination of genetic susceptibility and environmental factors, with ERVWE1 potentially acting as a bridging mediator. Its upregulation involves multiple layers of regulation, including environmental, genetic, epigenetic modification and others [81]. Environmental stressors such as viral infection [82,83] and certain medications [48] can induce ERVWE1 expression. Moreover, *ERVWE1* resides in the schizophrenia risk region 7q21, and genetic variants at this locus or its regulatory elements may modulate expression [44]. Epigenetic derepression, including upregulation of the demethylase ALKBH5 by ERVWE1 itself [59], can further enhance expression. Together, these factors likely converge, leading to ERVWE1 upregulation in genetically susceptible individuals exposed to environmental stress, thereby amplifying downstream pathogenic effects.

While our findings provide novel insights into the ERVWE1/miR-27b-3p/BNIP3 axis in schizophrenia, several limitations should be acknowledged. First, the link between ERVWE1 and BNIP3 is indirect and appears to be mediated through the suppression of miR-27b-3p. While our study provides solid evidence that miR-27b-3p directly targets BNIP3, the upstream mechanisms by which ERVWE1 leads to the downregulation of this specific miRNA remain incompletely defined. It is plausible that ERVWE1 activates broader cellular stress–responsive signaling pathways that secondarily alter miRNA transcription, processing, or stability. Elucidating these upstream regulatory events will be an important focus of future studies. Second, as with most case-control studies in psychiatric research, potential confounding effects cannot be fully excluded. Factors such as chronic psychological stress, comorbid inflammation states, and antipsychotic medication use may influence the expression of ERVWE1, miRNAs, or BNIP3. Future studies involving medication-naive patients, longitudinal designs, or detailed environmental and inflammatory profiling will be necessary to better isolate the disease-specific contribution of the ERVWE1–miR-27b-3p–BNIP3 axis. Thirdly, the mechanistic conclusions are primarily derived from in vitro models and bioinformatic analyses, limiting direct extrapolation to in vitro schizophrenia pathology. Future studies with animal models and neuropathological validation are needed to confirm the relevance and therapeutic potential of this axis.

## 5. Conclusions

**Figure 10:**
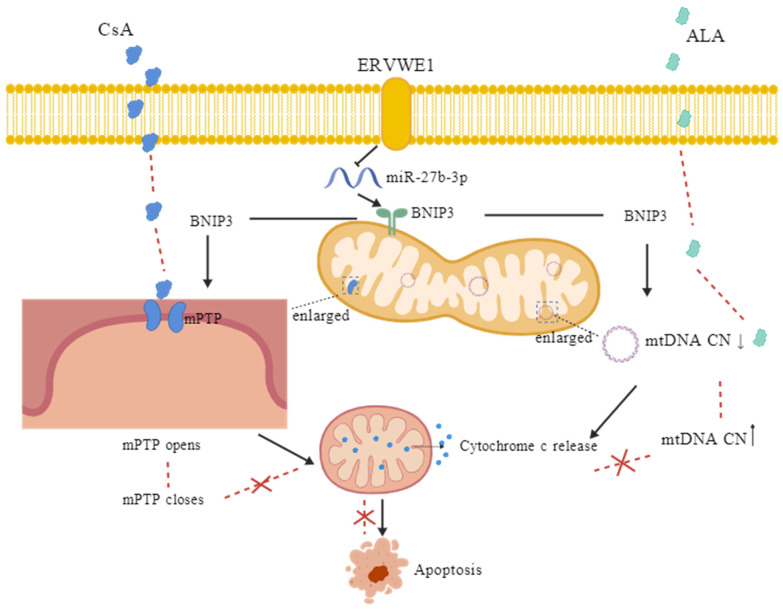
Schematic model illustrating ERVWE1-induced mitochondrial structural damage and apoptosis via the miR-27b-3p/BNIP3 signaling pathway. Arrows indicate the direction of regulation and the progression of pathological events. Specifically, arrows next to mtDNA CN denote its increase or decrease. Dashed lines indicate the effects following drug treatment (e.g., CsA, ALA); “×” indicates inhibition in the pathway.

Collectively, our findings support a model in which aberrant ERVWE1 activation disrupts mitochondrial homeostasis through miR-27b-3p-dependent upregulation of BNIP3, leading to mitochondrial structural damage, cytochrome c release, and neuronal apoptosis (Figure 10). This pathway provides a molecular framework linking endogenous retroviral activity to core cellular pathology in schizophrenia and offers new avenues for therapeutic intervention.

## Data Availability

The data that support the findings of this study are available from Gene Expression Omnibus at Home—GEO—NCBI (https://www.nih.gov/), reference numbers GSE53987.

## Supplementary Materials

The following supporting information can be downloaded at: https://www.mdpi.com/article/10.3390/v18020245/s1, Supplementary Figure S1: The transfection efficiency of ERVWE1 in SH-SY5Y cells. Supplementary Figure S2: The transfection efficiency of BNIP3 in SH-SY5Y cells. Supplementary Figure S3: The transfection efficiency of shBNIP3 in SH-SY5Y cells. Supplementary Figure S4: The transfection efficiency of miR-27b-3p mimic, ERVWE1 and miR-27b-3p mimic co-transfection in SH-SY5Y cells. Supplementary Table S1: Comparison of the plasma samples demographic data between the healthy controls and schizophrenia patients. Supplementary Table S2: Primer sequence for real-time PCR and cloning used in this study. Supplementary Table S3: Antibodies and the dilutions in Western blotting.
