# Supplementary material for: ERVWE1 Impairs Mitochondrial Homeostasis and Promotes Neuronal Apoptosis via the miR-27b-3p/BNIP3 Axis in Schizophrenia
Source: Viruses. 2026 Feb 14;18(2):245. doi: 10.3390/v18020245 (PMC12944911; doi:10.3390/v18020245)
Supplement: Supplementary file 1 [file viruses-18-00245-s001.zip › viruses-4102536-supplementary.pdf]

# Supplementary Materials

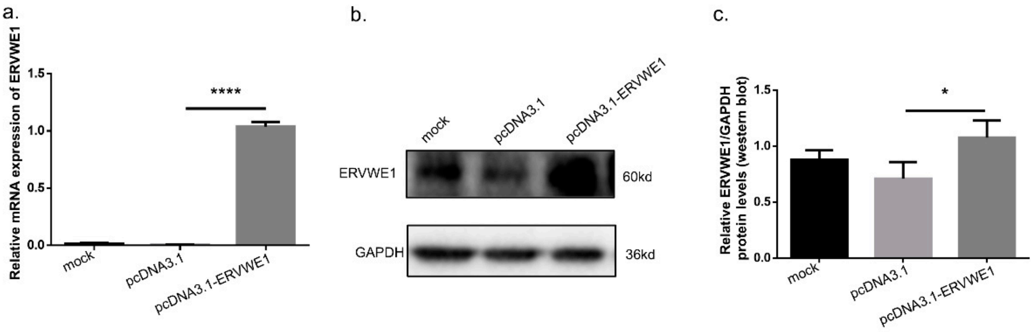


**Supplementary Figure S1** The transfection efficiency of ERVWE1 in SH-SY5Y cells. a RT-qPCR analysis of ERVWE1 mRNA expression levels in SH-SY5Y cells transfected with ERVWE1. b-c Western blotting analysis confirming ERVWE1 protein expression in ERVWE1-transfected SH-SY5Y cells. Statistical analysis: one-way ANOVA (* $p$ <0.05 and **** $p$ < 0.0001)

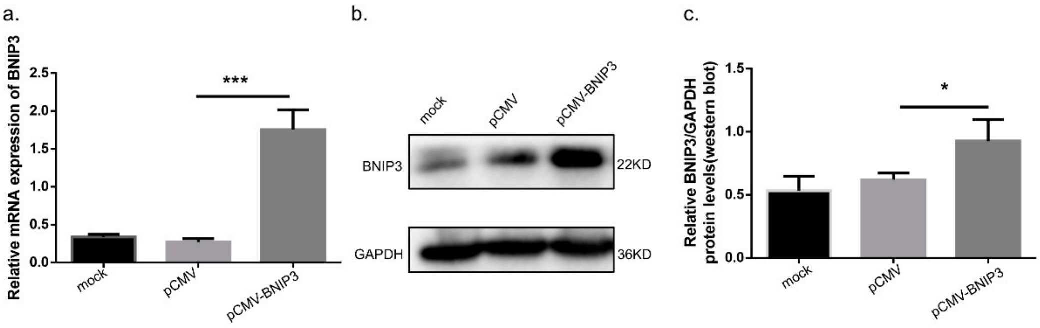


**Supplementary Figure S2** The transfection efficiency of BNIP3 in SH-SY5Y cells. a RT-qPCR analysis of BNIP3 mRNA expression levels in SH-SY5Y cells transfected with BNIP3. b-c Western blotting analysis confirming BNIP3 protein expression in BNIP3-transfected SH-SY5Y cells. Statistical analysis: one-way ANOVA (* $p$ < 0.05 and *** $p$ < 0.001)

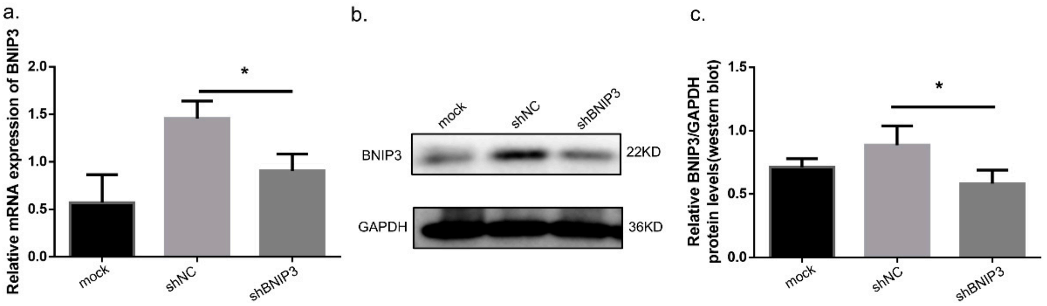


**Supplementary Figure S3** The transfection efficiency of shBNIP3 in SH-SY5Y cells. a RT-qPCR analysis of BNIP3 mRNA expression levels in SH-SY5Y cells transfected with shBNIP3. b-c Western blotting analysis confirming BNIP3 protein expression in shBNIP3-transfected SH-

SY5Y cells. Statistical analysis: one-way ANOVA (* $p < 0.05$)

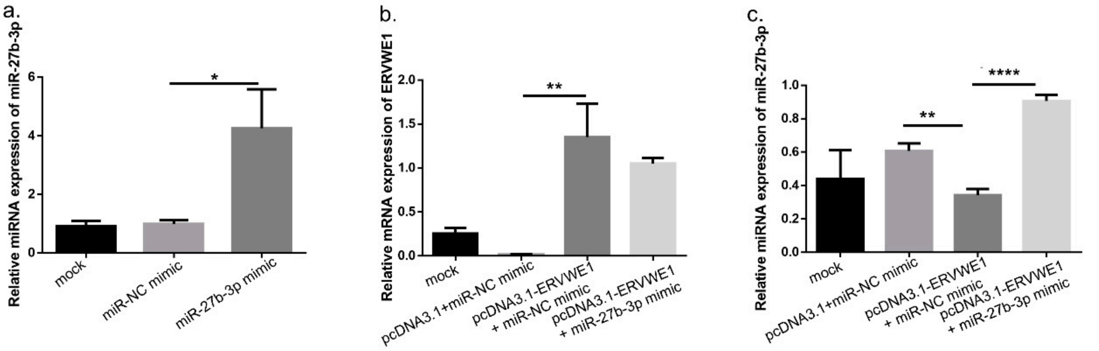


**Supplementary Figure S4** The transfection efficiency of miR-27b-3p mimic, ERVWE1 and miR-27b-3p mimic co-transfection in SH-SY5Y cells. a RT-qPCR analysis of miR-27b-3p expression levels in SH-SY5Y cells. b RT-qPCR analysis of ERVWE1 mRNA expression levels in SH-SY5Y cells co-transfected with ERVWE1 and miR-27b-3p mimic. c RT-qPCR analysis of miR-27b-3p mRNA expression levels in SH-SY5Y cells co-transfected with ERVWE1 and miR-27b-3p mimic. Statistical analysis: one-way ANOVA (* $p < 0.05$, ** $p < 0.01$ and **** $p < 0.0001$)

**Supplementary Table S1.** Comparison of the plasma samples demographic data between the healthy controls and schizophrenia patients.

| | Schizophrenia (n = 44) | | Controls (n = 44) | | Analysis |
|---|---|---|---|---|---|
| | Median | Range | Median | Range | *P* |
| Age (year)[a] | 39 | 25–63 | 42 | 23–59 | 0.5761 |
| Education (year)[a] | 11 | 3–16 | 12 | 6–16 | 0.4412 |
| BMI (body mass index)[a] | 21.0 | 15.5–26.3 | 21.95 | 17.4–25.8 | 0.3131 |
| | N | % | N | % | *P* |
| Gender[b] | | | | | |
| Male | 20 | 47 | 23 | 53 | 0.5233 |
| Femal | 24 | 53 | 21 | 47 | |
| Smoking status[b] | | | | | |
| Yes | 18 | 41 | 15 | 34 | 0.5089 |
| No | 26 | 59 | 29 | 66 | |

Notes:

[a] *P* values were calculated by the Mann-Whitney *U* test.

[b] *P* values were calculated by the chi-square test.

**Supplementary Table S2.** Primer sequence for real-time PCR and cloning used in this study.

| Target gene | Oligo sequence (5' > 3') |
|---|---|
| ERVWE1-F | CCAATGCATCAGGTGGGTAAC |
| ERVWE1-R | GAGGTACCACAGACAAAAAATATTCCT |
| GAPDH-F | GGAGCGAGATCCCTCCAAAAT |

| | |
|---|---|
| GAPDH-R | GGCTGTTGTCATACTTCTCATGG |
| BNIP3-F | CAGGGCTCCTGGGTAGAACT |
| BNIP3-R | CTACTCCGTCCAGACTCATGC |
| hsa-miR-27b-3p | GTCGTATCCAGTGCAGGGTCCGAGGTATTCGCACTGGATACGACGCAGAA |
| hsa-miR-27b-3p-F | CGGCCGCGTTCACAGTGGCTAAG |
| hsa-miR-27b-3p-R | GCAGGGTCCGAGGTATTC |
| RNU6-1-F | CTCGCTTCGGCAGCACA |
| RNU6-1-R | AACGCTTCACGAATTTGCGT |
| BNIP3 3-UTR WT-F | CTAGCCAGACTGATTAATGTCTACTGTGAAAATTTGGTAGTAAATTTTCATTTGAT |
| BNIP3 3-UTR WT-R | CTAGATCAAATGAAAATTTACTACCAAATTTTCACAGTAGACATTAATCAGTCTGG |
| BNIP3 3-UTR mut-F | CTAGCCAGACTGATTAATGTCTGACTCATAAATTTGGTAGTAAATTTTCATTTGAT |
| BNIP3 3-UTR mut-R | CTAGATCAAATGAAAATTTACTACCAAATTTATGAGTCAGACATTAATCAGTCTGG |
| shNC-F | GATCCGCGGGAAATCATACCCTTACCCGAAGGTAAGGGTATGATTTCCCGCA |
| shNC-R | AGCTTGCGGGAAATCATACCCTTACCTTCGGGTAAGGGTATGATTTCCCGCG |
| shbnip3-F | GATCCGAACTGCACTTCAGCAATAATCGAAATTATTGCTGAAGTGCAGTTCA |
| shbnip3-R | AGCTTGAACTGCACTTCAGCAATAATTTCGATTATTGCTGAAGTGCAGTTCG |
| hsa-miR-27b-3p mimics-F | UUCACAGUGGCUAAGUUCUGC |
| hsa-miR-27b-3p mimics-R | AGAACUUAGCCACUGUGAAUU |
| mimic-NC-F | UUUGUACUACACAAAAGUACUG |
| mimic-NC-R | CAGTACTTTTGTGTAGTACAAA |

**Supplementary Table S3**.Antibodies and the dilutions in Western blotting

| Antibodies | Brands | Catalogs | Dilutions |
|---|---|---|---|
| ERVWE1 Rabbit pAb | Abclonal | A16522 | 1:1,000 |
| GAPDH Mouse mAb | Abclonal | AC002 | 1:10,000 |
| BNIP3 Rabbit pAb | Abclonal | A5683 | 1:1,000 |
| DRP1 Rabbit mAb | Abclonal | A21968 | 1:5,000 |
| Cytochrome C Rabbit mAb | Abclonal | A4912 | 1:10,000 |
| VDAC1 Rabbit mAb | Abclonal | A19707 | 1:4,000 |
